# Anti-OX40 Biological Therapies in the Treatment of Atopic Dermatitis: A Comprehensive Review

**DOI:** 10.3390/jcm13226925

**Published:** 2024-11-17

**Authors:** Myriam Marfil-Cantón, Alvaro Prados-Carmona, Marta Cebolla-Verdugo, Husein Husein-ElAhmed, Fernando Campos, Ricardo Ruiz-Villaverde

**Affiliations:** 1 Dermatology Department, Hospital Universitario San Cecilio, 18016 Granada, Spain; myriam.marfil.sspa@juntadeandalucia.es (M.M.-C.); alvaro.prados.sspa@juntadeandalucia.es (A.P.-C.); marta.cebolla.sspa@juntadeandalucia.es (M.C.-V.); husein.husein.sspa@juntadeandalucia.es (H.H.-E.); 2 Instituto Biosanitario de Granada, Ibs, 18012 Granada, Spain; fcampos@ugr.es; 3 Dermatology Department, Hospital Universitario de Baza, 18800 Granada, Spain; 4 Tissue Engineering Group, Department of Hisotology, University of Granada, 18016 Granada, Spain

**Keywords:** monoclonal antibody, atopic dermatitis, biological therapy, inflammation, OX40, OX40L

## Abstract

**Introduction.** Atopic dermatitis (AD) is the most prevalent inflammatory dermatological disorder, affecting a significant percentage of the global population. This chronic disease has a multifactorial and intricate pathogenesis, influenced by genetic predisposition, skin barrier dysfunction, immune dysregulation, neuroimmune mechanisms, and alterations in the skin microbiome, among other factors. **Methods.** The treatment of AD has faced significant clinical challenges due to the ineffectiveness of conventional therapies. However, recent advances in understanding its pathophysiology have led to the introduction of new therapeutic options. Recently, the OX40 receptor has been identified as a key factor in the development of AD. Recent studies have demonstrated that blocking the OX40 ligand with monoclonal antibodies significantly and sustainably improves the signs and symptoms of moderate to severe AD. **Results.** A comprehensive review of the available literature on anti-OX40 treatments in atopic dermatitis that evaluates their mechanism of action, their clinical efficacy, and the prospects of this promising therapeutic option for improving AD management is provided. **Conclusions.** Anti-OX40 and anti-OX40L blockers are a promising therapeutic alternative for the management of moderate–severe atopic dermatitis. Prospective analytical studies are needed to determine whether this new therapeutic target represents a qualitative advance in modifying the progression of the disease.

## 1. Introduction

Atopic dermatitis (AD) is a widely acquired recurrent inflammatory skin disease. This chronic ailment is characterized by a recurring inflammation of the skin and a complicated interaction between skin barrier dysfunction and T-cell-driven inflammation. Affecting 2.1–4.9% of adults and up to 20% of children, AD’s prevalence continues to rise worldwide, with heterogeneous endophenotypes and a high disease burden [1,2].

The diagnostic criteria for atopic dermatitis are based on the clinical evaluation of characteristic signs and symptoms of the disease (Hanifin and Rajka diagnostic criteria). Clinically, AD presents a wide range of variable symptoms, such as skin inflammation, eczema, barrier disruption, erythema, papules and vesicles, excoriations, lichenification, and intense pruritus, which often worsens at night, leading to sleep disturbances. Abnormal microbial colonization, notably by *Staphylococcus aureus*, is also linked to barrier disruption [3].

These symptoms lead to discomfort, stress, and overall reduced quality of life.

The severity of AD symptoms and their impact on daily activities are assessed using several standardized measurement scales. These scales include both objective and subjective parameters, providing a comprehensive view of the disease and its effect on the patient’s quality of life [4,5].

This review aims to discuss the OX40–OX40L pathway as a potential therapeutic target for AD, providing a comprehensive overview of the pathogenesis and therapeutic advancements in managing this complex disease.

### 1.1. Objective Parameters

The Eczema Area and Severity Index (EASI) is a tool used to quantify eczema severity by evaluating four body areas: head/neck, trunk, upper limbs, and lower limbs. It combines the extent and intensity of symptoms (redness, thickness, scratching, and lichenification) to provide a score from 0 (no eczema) to 72 (most severe).

The SCORing Atopic Dermatitis (SCORAD) index assesses both the extent and severity of eczema, considering clinical signs (redness, swelling, and oozing) and patient-reported symptoms (itching, sleep loss, etc.). The score ranges from 0 to 103, with higher scores indicating more severe disease.

### 1.2. Subjective Parameters

The Numeric Rating Scale (NRS) for pruritus measures itching intensity, with patients rating their itchiness from 0 (no itching) to 10 (worst possible itching).

The Visual Analog Sleep Scale (VAS) evaluates the patient’s sleep quality using a 10 cm horizontal line, where 0 represents “the worst possible sleep” and 10 “the best possible sleep”.

The Patient-Oriented Eczema Measure (POEM) is a seven-question questionnaire covering common symptoms of eczema such as itching, disturbed sleep, dry skin, peeling, cracking, redness, and bleeding. Each question has a five-point scale (0 to 4), and the total score ranges from 0 to 28, with higher scores indicating greater severity of symptoms.

These scales together offer a comprehensive assessment of AD severity and its effects, guiding treatment decisions and monitoring outcomes.

Although there have been approaches to improve the diagnostic criteria (Hanifin’s criteria are still valid since 1980) and homogenization of the monitoring of the severity index by the previously mentioned scales, it is within AD therapy where a real emergence has occurred in the last 6 years [6].

According to the latest published European guidelines based on consensus for the conventional management of atopic eczema (AD) in adults and children, the different lines of treatment for atopic dermatitis are described in the following table.

Additionally, even more promising treatments are currently in development. The diverse clinical responses observed in both clinical trials and real-world scenarios, along with the economic implications, underscore the importance of precision medicine. Major challenges include identifying which patients will respond to specific treatments, gaining a deeper understanding of disease modification and comorbidities in AD, and optimizing therapeutic strategies [7,8]. Essential goals include preventing the onset, progression, and chronicity of AD and managing associated comorbidities through immunomodulatory therapies, complemented by emollients, patient education, and lifestyle modifications such as regular exercise and smoking cessation [3,9].

Despite significant advancements, treating AD remains challenging due to its heterogeneity and unpredictable disease course. Recent discoveries in the pathophysiology of AD have identified new targeted therapies, broadening the range of treatment options available to patients [2,10].

The pathophysiology of AD is complex and characterized by barrier dysfunction and abnormal immune responses. Mutations have been identified that cause reduced levels of filaggrin (FLG), a protein essential for the integrity of the skin barrier. These FLG deficiencies are exacerbated by the type 2 immune response, in which interleukins IL-4 and IL-13 play a crucial role by inhibiting FLG expression [11]. Additionally, keratinocytes in the affected skin amplify this immune response by producing epithelial cytokines such as thymic stromal lymphopoietin (TSLP), IL-25, and IL-33 [10]. These cytokines promote inflammation and perpetuate the cycle of barrier dysfunction and exacerbated immune response, leading to the characteristic symptoms of AD [1,12].

Cytokines such as interleukin (IL)-4 and IL-13 have been identified as key contributors to the allergic inflammation associated with this presentation [11,13]. The crucial role of these two cytokines has been highlighted by the strong response to treatment with the anti-IL-4 receptor alpha (IL4R) antibody dupilumab, which blocks the IL-4 and IL-13 signaling pathways [1,12].

The IL-13 pathway can also be specifically blocked by other antibodies (lebrikizumab and tralokinumab) [7].

Dupilumab and tralokinumab are the only monoclonal antibodies currently approved for the treatment of moderate to severe AD in adults (Table 1) [2,8].

Moreover, IL-31 produced by Th2 cells, is believed to mediate atopic itch. Regarding this, anti-IL-31 receptor antibody nemolizumab has shown remarkable effectiveness in reducing pruritus in AD patients shortly after administration [2,7,12].

Other innovative treatments include oral inhibitors of Janus kinase (JAK), another pathway that has been shown to be involved in the pathogenesis of AD [7]: Baricitinib (JAK1,2 inhibitor), Upadacitinib, and Abrocitinib (specific JAK1 inhibitors) [2,8].

Here are named novel targets such as OX40 (tumor necrosis factor receptor superfamily member 4) and its ligand OX40L (tumor necrosis factor ligand superfamily member 4). These targets hold promise by potentially affecting multiple immune pathways critical to AD pathogenesis, offering effective disease control regardless of the subtype or stage [14,15].

OX40 acts like a T-cell costimulatory molecule. This molecule is highly expressed on activated T cells in patients suffering from AD. The link between OX40 and its ligand (OX40L) facilitates the proliferation, specialization, and persistence of harmful effector and memory T cells [9,16]. Encouraging early-phase clinical trial outcomes for anti-OX40 antibodies (rocatinlimab and telazorlimab) and the anti-OX40L antibody (amlitelimab) in moderate to severe AD highlight the promise of targeting the OX40 pathway as an innovative treatment strategy for AD [13,14].

## 2. Material and Methods

Study design: A bibliographic review was carried out focusing on searches for research articles on anti-OX40 and anti-OX40L related to ongoing clinical trials and review articles on their pathophysiological and clinical implications in the period between May and September 2024.

Search strategy: A search was carried out in the main databases available on the Internet at an international level: PubMed, Scopus, WOS, Scielo, and Google Scholar as well as others at the Spanish level, such as Dialnet and Ibecs.

Critical evaluation of the selected articles: The selected articles were read to determine whether or not they were suitable for inclusion in the bibliographic review. They were analyzed, checking that they contained and presented the main findings that were included in the current review.

## 3. Atopic Dermatitis Pathogenesis and the Role of OX40–OX40L Pathway

### 3.1. Atopic Dermatitis Pathogenesis

Although it was historically believed that AD was solely caused by defects in the epithelial physical barrier leading to excessive T-cell activation, emerging evidence now indicates that this recurrent condition involves more components of the immune system as well as other elements such as the epithelium. Besides being the outermost layer of the skin and being responsible for the protective function, the epithelium plays a crucial role in the sensitization process of Th2 cells through its stimulating effects on dendritic cells (DC) [15].

Today, it is known that AD is characterized by inflammation caused by both innate and adaptive immune cells and various chemokines and cytokines. Th2 helper cells play a fundamental role by producing ILs such as IL-4 and IL-13, which induce the production of thymic stromal lymphopoietin (TSLP) in the keratinocytes of skin lesions, playing a significant role in AD pathogenesis. The relationship between AD pathogenesis and TSLP is implied by its high expression in the keratinocytes of AD patients and in skin samples subjected to barrier disruption by tape stripping. In AD, serum TSLP levels significantly increase [1,12,15].

Moreover, the increased production of TSLP in keratinocytes is promoted by the microorganism *Staphylococcus aureus*, present in the skin biome [7,12].

TSLP also modulates DC polarization by increasing OX40L expression. Activated DCs expressing OX40L interact with OX40 on naïve helper T cells, leading to Th2 cell proliferation and cytokine production [15,17].

These cytokines activate signal transducers STAT6 and STAT3, negatively regulating essential proteins for skin barrier function, such as filaggrin (FLG), loricrin (LOR), and involucrin (IVL). Besides IL-4 and IL-13, other cytokines like IL-22, IL-31, and IL-17 also contribute to skin barrier disruption [1].

In addition, AD frequently involves elevated levels of IgE in the blood plasma and an increase in circulating activated T cells as well as high levels of L-selectin, a leukocyte activation marker that indicates disease severity [15].

Overall, both TSLP and OX40 are crucial cytokines in the activation and Th2 skewing of the immune system in AD [7].

### 3.2. Mechanism of OX40/OX40L in the Pathogenesis of Atopic Dermatitis

Recent research has focused on the potential role of OX40/OX40L signaling in atopic dermatitis (AD). The molecule OX40 and its ligand OX40L play a crucial role in the pathogenesis of AD. OX40 (CD134) is a costimulatory molecule belonging to the tumor necrosis factor receptor (TNFR) family, predominantly expressed on T cells following their activation. OX40L, its ligand, is found on activated antigen-presenting cells, such as dendritic cells and endothelial cells [1,8,14].

Although the expression of OX40 and OX40L is not limited to T cells and dendritic cells, these markers are also found in endothelial cells and smooth muscle cells. This can lead to adverse events both at the sites of inflammation and in other tissues during therapeutic interventions. Therefore, while therapies targeting OX40 and OX40L are promising, it is crucial to consider their potential systemic side effects [9,17].

**Figure 1:**
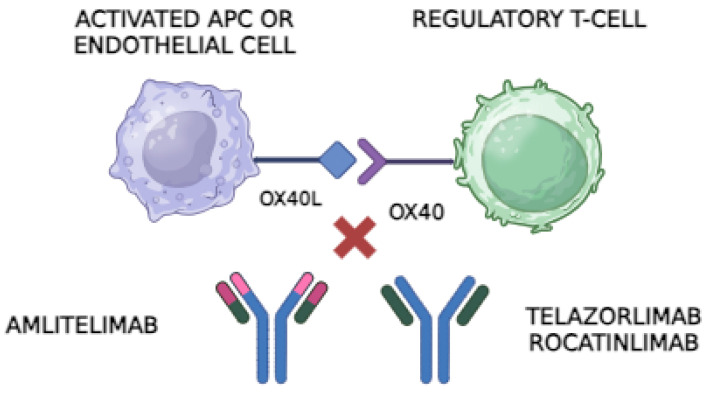
The OX40 receptor, found on regulatory T cells (Tregs) and other activated T cells, binds to its ligand, OX40L, on antigen-presenting cells (APCs) or endothelial cells. This interaction promotes T-cell activation and proliferation, contributing to inflammatory responses. Anti-OX40 antibodies, such as Telazorlimab and Rocatinlimab, block the OX40 receptor on T cells, preventing it from interacting with OX40L. This interruption reduces T-cell activation and can limit the inflammatory cascade in autoimmune diseases. In contrast, anti-OX40L antibodies like Amlitelimab bind directly to OX40L on APCs, blocking its interaction with OX40 on T cells. This mechanism similarly inhibits T-cell activation but without directly targeting the T-cell population, potentially modulating the immune response while preserving T-cell numbers. Both types of antibodies are being explored as treatments for conditions like atopic dermatitis due to their potential to reduce inflammation and improve symptoms in patients with immune-mediated diseases. Image created through Biorender^®^.

The interaction between OX40 and OX40L is essential for the amplification of T-cell-mediated immune responses. This interaction not only promotes the proliferation and prolonged survival of T cells by suppressing apoptosis but also enhances their ability to produce cytokines and form CD4+ memory T cells, resulting in a stronger and more durable immune response. In the context of AD, this signaling contributes to the perpetuation of chronic inflammation and skin barrier dysfunction (Figure 1) [8,9,13].

In autoimmunity, the upregulation of OX40-OX40L plays a crucial multifaceted role in disrupting T-cell tolerance. In patients with AD, there is a notable overexpression of OX40 in T cells and an increased number of dendritic cells expressing OX40L [2,16]

Furthermore, OX40 is typically upregulated at sites of inflammation, particularly in AD lesions, where it is prominently found in infiltrating lymphocytes and circulating peripheral lymphocytes [9].

The increased OX40-OX40L signaling is associated with the following [2,9,13,14,15,16,17]:

Activation and expansion of T helper type 2 (Th2) cells: This is crucial in the pathogenesis of AD.

Impairment of the suppressive functions of regulatory T cells (Tregs), contributing to an uncontrolled and chronic inflammatory response: In these cells, the Foxp3 gene has altered their suppressive functions. This disruption of immune tolerance is a key component in disease progression, as Tregs normally act to maintain immune tolerance and prevent excessive autoimmune responses.

Bridging of Th2 and Th1 pathways by inducing the release of IFN-gamma, a cytokine typical of the Th1 response: This can result in the conversion of autoreactive T cells into effector T cells, exacerbating inflammation. Compared to normal skin, AD lesions show upregulation of the OX40/OX40L axis, indicating increased inflammatory activity in these sites.

## 4. Anti-OX40 Treatments

The importance of the OX40–OX40L pathway in AD has driven the development of new specific therapies for this target. Initial clinical studies with antibodies directed against OX40 and its ligand OX40L have shown encouraging results in decreasing inflammation and reducing symptoms of atopic dermatitis. These therapies seek to block the interaction between OX40 and OX40L, leading to reduced activation and proliferation of Th2 cells, thereby reducing the production of inflammatory cytokines and improving the barrier function of the skin. These findings suggest a high therapeutic potential for these new treatment strategies in the management of this disease.

### Development and Mechanisms of Action: How Anti-OX40 Treatments Act

The inhibition of the OX40 pathway can be carried out in two different ways: either by binding to the OX40 receptor or by binding to the ligand of this receptor, OX40L. The outcome remains the same: the blockade of the OX40–OX40L interaction. Based on this, we can distinguish between two groups within the anti-OX40/OX40L treatments.

## 5. Anti-OX40 Antibodies

### 5.1. Telazorlimab (GBR 830)

Telazorlimab, initially known as GBR 830, was the first antibody targeting OX40 that was evaluated in a phase 2a trial, where its efficacy, safety, and adverse effects were investigated in patients with AD (atopic dermatitis) [8]. Telazorlimab is a humanized monoclonal IgG1 antibody whose mechanism of action involves binding to OX40, a co-stimulatory receptor present on activated T cells, and inhibiting it. This mechanism makes it particularly promising for the treatment of chronic autoimmune inflammatory disorders, including AD. As an OX40 antagonist, it blocks the signaling mediated by this receptor, preventing T-cell activation and proliferation, which in turn reduces the inflammatory cascade characteristic of diseases mediated by these cells, such as AD. Moreover, it not only suppresses T-cell activation but also decreases the production of pro-inflammatory cytokines [1,9,13].

The development of telazorlimab represents a significant advance in therapeutics, as it is the first OX40 antagonist to have completed phase I studies globally and is currently in phase II clinical trials, still recruiting patients (NCT02683928). These trials are assessing its efficacy and safety in a larger number of patients with AD [1,7,9].

The study demonstrated that telazorlimab, administered subcutaneously, was effective in improving symptoms of moderate to severe atopic dermatitis. Additionally, in terms of efficacy and safety, the drug was well tolerated both in single and multiple doses administered intravenously at doses up to 40 mg/kg, with a similar distribution of adverse events between the treatment and placebo groups [9,14].

In the treated patients, a significant reduction was observed in the mRNA levels of biomarkers associated with the TH1, TH2, TH17, and TH22 pathways, suggesting a positive impact on both the acute and chronic phases of the disease. Specifically, there was a decrease in the number of OX40+ T cells and OX40L+ dendritic cells, which are crucial in TSLP-mediated inflammation [9,13].

Besides its safety profile, telazorlimab has shown promising efficacy in reducing AD symptoms. In a phase 2a trial, 76.9% of patients treated with telazorlimab achieved a significant improvement, as measured by the EASI-50, compared to only 37.5% in the placebo group. This result underscores its effectiveness in the clinical improvement of AD. Additionally, a significant reduction was observed in the levels of cytokines such as IL-31, CCL11, CCL17, and S100 in treated patients [7,14,16].

The results suggest that telazorlimab could be a promising option for the treatment of AD, as it showed remarkable improvements compared to other biological treatments and JAK inhibitors. However, additional long-term studies with a larger number of patients are needed to confirm its safety and efficacy in moderate to severe AD [7,8,9,13].

### 5.2. Rocatinlimab

Rocatinlimab, previously known as AMG 451/KHK4083, is a fully human anti-OX40 immunoglobulin G1 (IgG1) monoclonal antibody. This antibody specifically works by depleting OX40+-activated T cells and suppressing the clonal expansion of T cells, suggesting its potential to control conditions driven by the Th2-type immune response [13,16,18].

In a phase 1 clinical trial, 22 patients with moderate to severe atopic dermatitis (AD) received intravenous rocatinlimab at a dose of 10 mg/kg every two weeks over a six-week treatment period, followed by a 16-week follow-up period. The results indicated that infusion-related adverse reactions were mostly mild to moderate in severity, with the most common being pyrexia (50%) and chills (36.4%). Other adverse effects included aphthous ulcers, increased blood uric acid, nasopharyngitis, erythema, and styes. Despite these side effects, a significant reduction in the EASI score was observed, with an average decrease of 74.12% by day 155, suggesting a notable clinical efficacy of rocatinlimab in managing AD [13,16,18].

Subsequently, a phase 2b, multicenter, randomized, double-blind, placebo-controlled trial evaluated the efficacy and safety of rocatinlimab in 274 patients with moderate to severe AD who had shown an inadequate response to conventional topical treatments. Patients were assigned to one of five treatment groups, receiving different doses of subcutaneous (SC) rocatinlimab or placebo over 36 weeks, followed by a 20-week drug-free follow-up period. The results showed that all groups treated with rocatinlimab experienced significant reductions in the EASI score at week 16 compared to the placebo group. Moreover, a considerable proportion of patients achieved a 75% improvement in the EASI score (EASI75), with responses maintained even after discontinuation of treatment [1,18].

A more detailed study of the effects of rocatinlimab included transcriptomic and proteomic analyses of skin biopsy and serum samples from patients, revealing that treatment with rocatinlimab led to a significant reduction in OX40 mRNA expression and downregulation of genes related to the Th2, Th1/Th17, and Th22 pathways. These molecular changes persisted even after treatment discontinuation, suggesting that rocatinlimab not only is effective in reducing AD symptoms but may also have lasting effects that modify the disease course [16,18].

Regarding safety, up to 81% of patients treated with rocatinlimab in this clinical trial experienced adverse events during the study, with the most common being pyrexia, chills, and nasopharyngitis. These effects were mostly mild to moderate and occurred mainly after the first administration of the drug. No deaths were reported, and serious adverse events were rare and not directly related to the treatment [18].

Preliminary results from these studies are promising, indicating that rocatinlimab could be an effective and safe therapeutic option for patients with moderate to severe AD, especially those who do not adequately respond to existing treatments. Phase 3 studies are currently underway to further evaluate the efficacy, safety, and tolerability of rocatinlimab as monotherapy and in combination with other treatments, aiming to establish its role in the clinical management of AD [13,16] Continued clinical trials will be crucial to confirm these findings and determine its application in clinical practice.

The difference between rocatinlimab and telazorlimab is that rocatinlimab is an afucosylated anti-OX40 antibody that exhibits enhanced antibody-dependent cellular cytotoxicity (ADCC), whereas telazorlimab specifically blocks OX40 signaling in activated T cells with moderate levels of ADCC (Ichnos Sciences, unpublished data) [14].

## 6. Anti-OX40L Antibodies

### Amlitelimab

Amlitelimab, also known as KY1005 or SAR445229, is a novel human IgG4 monoclonal antibody used in the treatment of atopic dermatitis (AD). Unlike treatments that block the OX40 receptor on T cells, such as rocatinlimab and telazorlimab, amlitelimab works by binding to the ligand of this receptor, OX40L, on antigen-presenting cells (APCs), preventing the interaction between OX40L and OX40. This blockade reduces T-cell activation and the production of pro-inflammatory cytokines. Additionally, its ability to modulate the immune response without depleting T cells suggests that it could offer significant benefits in managing chronic and inflammatory diseases [1,13,17].

Amlitelimab has previously been evaluated in studies aimed at determining its safety and efficacy. A phase 1 trial conducted with 64 healthy subjects showed that amlitelimab is well tolerated, with mainly mild and self-limiting adverse events [16].

Initial trials have demonstrated that amlitelimab is effective in reducing the symptoms of AD, positioning itself as a promising therapeutic option for patients who do not respond adequately to conventional topical treatments.

In a phase 2a study that included 89 patients with moderate to severe AD, amlitelimab showed a significant clinical response in just two weeks. By week 16, patients treated with both low and high doses of amlitelimab achieved reductions in EASI scores of 80.1% and 69.9%, respectively, compared to a 49.4% reduction in the placebo group. Moreover, a considerable percentage of patients achieved a 75% improvement in their EASI scores, with 59.3% in the low-dose group and 51.9% in the high-dose group [1,14,16,19].

Notably, 68% of patients who responded to the treatment maintained their improvement for 24 weeks after the last dose, suggesting a long-lasting and sustained effect. Additionally, the incidence of adverse events was low and comparable between the treatment and placebo groups, supporting its safety profile [16].

Beyond the clinical benefits mentioned, a significant reduction in IL-22 levels, a cytokine involved in the pathogenesis of AD, was observed, suggesting a mechanism of action that affects multiple inflammatory pathways [17,19].

Currently, amlitelimab is being evaluated in a phase 2b trial to confirm its safety and efficacy in a larger number of patients with moderate to severe AD, which could establish it as a key treatment for the long-term management of AD. Furthermore, amlitelimab is being investigated for the treatment of other immune-mediated diseases, such as moderate to severe asthma, which broadens its therapeutic potential [1,17].

In Table 2, we summarize the main results of ongoing clinical studies on anti-OX40 and anti-OX40L drugs, as previously discussed.

## 7. Conclusions

These new biological treatments represent an advanced and promising therapeutic option for the treatment of atopic dermatitis and possibly other immune-mediated conditions, offering a favorable safety profile and sustained clinical effects.

The value of therapeutic agents targeting OX40 lies in their ability to avoid affecting naive and resting memory T cells. These two distinctive characteristics of their mechanism of action, which target TH2-skewed immune responses without triggering naive and memory T cells, may help explain the clinical efficacy and safety profiles of OX40-targeted drugs, including telazorlimab and rocatinlimab.

## Figures and Tables

**Table 1:** Lines of treatment for atopic dermatitis, based on the document of Consensus [6].

Type	Examples	Mechanism of Action	Phase	Observations
Topical Treatment	Corticoids	Anti-inflamatory	Mild–moderate	Long-term adverse effects
	Calcineurin inhibitors (tacrolimus and pimecrolimus)	Immunosuppressant	Mild–moderate	Long-term adverse effects
	Emollients	Reinforcement of the barrier function	Mild	Reduces symptoms but does not remove signs.
Systemic	Oral corticoids	Anti-inflamatory	Moderate–severe	No indications by technical sheet for use in AD
	Cyclosporine	Immunosuppressant	Severe	It is used obligatorily
	Methotrexate	Immunosuppressant	Severe	MTX is used by extended indication
	Azathioprine	Immunosuppressant	Severe	It is used by extended indication
Biological	Dupilumab	Anti-IL4	Moderate–severe	Approved: FDA: March 2017 EMA: September 2017
	Tralokinumab Lebrikizumab	Anti-IL13	Moderate–severe	Approved: FDA: December 2021 EMA: June 2021 FDA: October 2023 EMA: September 2023
	Nemolizumab	Anti-IL31	Moderate–severe	To date, it has not received full approval from FDA or EMA clinical trials (phase III)
	Rocatinlimab telazorlimab	Anti-OX40	Moderate–severe	Phase III and II (clinical trials)
	Amlitelimab	Anti-OX40L	Moderate–severe	Phase II (clinical trials)
JAK Inhibitors	Baricitinib	Anti-Jak 1,2	Severe	Approved: FDA: May 2022 EMA: October 2020
	Upadacitinib	Anti-Jak 1	Severe	Approved: FDA: January 2022 EMA: August 2021
	Abrocitinib	Anti-Jak 1	Severe	Approved: FDA: January 2022 EMA: December 2021
Phototherapy	NB UVB, PUVA	Reduces inflammation and suppresses the immune system through exposure to UV light	Effective in some patients, non-pharmacological option	Not usually used as individual therapy

FDA: Food and Drug Administration; EMA: European Medicines Agency; AD: atopic dermatitis; JAK: Janus kinases; NB UVB: narrow band UVB; PUVA = psoralens + UVA; MTX: methotrexate

**Table 2:** Summary table of the main findings from studies conducted on anti-OX40 and anti-OX40L drugs.

Antibody	Clinical Trial Phase	Condition Studied	Mechanism of Action	Efficacy Results	Safety and Adverse Events	Additional Notes
**Telazorlimab [14]**	Phase 2a (ongoing)	Atopic dermatitis (AD)	Blocks OX40 receptor on T cells, inhibiting T-cell activation and cytokine production.	76.9% of patients achieved a significant improvement in EASI-50 score vs. 37.5% in placebo; reduced levels of cytokines (IL-31, CCL11, CCL17, and S100) and mRNA biomarkers related to TH1, TH2, TH17, and TH22 pathways.	Well tolerated in doses up to 40 mg/kg; adverse events comparable between treatment and placebo groups.	First OX40 antagonist to complete phase I trials and is now in phase II trials for AD; potential for other autoimmune diseases.
**Rocatinlimab [17]**	Phase 2b	Atopic dermatitis (AD)	Depletes OX40+ T cells and suppresses T-cell expansion, focusing on Th2-mediated immune responses.	74.12% decrease in EASI score by day 155 in phase 1; in phase 2b, a 75% EASI score improvement (EASI75) was sustained post treatment; reduced OX40 mRNA and Th2/Th1/Th17/Th22 pathway genes.	Up to 81% of patients experienced mild to moderate adverse events (pyrexia, chills, and nasopharyngitis); adverse events mostly after the first dose.	Ongoing phase 3 trials to evaluate as a standalone and combination therapy for AD; durable effects observed after treatment cessation.
**Amlitelimab [18]**	Phase 2b (ongoing)	Atopic dermatitis (AD)	Blocks OX40L on APCs, preventing T-cell activation by inhibiting OX40-OX40L interaction; modulates immune response without depleting T cells.	In phase 2a, low and high doses resulted in 80.1% and 69.9% EASI reductions at 16 weeks vs. 49.4% in placebo; 75% EASI improvement in 59.3% (low dose) and 51.9% (high dose) maintained in 68% of responders.	Low incidence of adverse events, comparable to placebo; mild, self-limiting side effects in phase 1 studies.	Potential for use in other immune-mediated diseases (e.g., asthma); durable clinical effect post treatment in AD with reduced IL-22 levels.
